# Artificial intelligence advancements in monoclonal antibody development technology

**DOI:** 10.3389/fimmu.2026.1802038

**Published:** 2026-06-25

**Authors:** Manar Ammar, Mikhail Samsonov, Evgeniya Gurylina, Daniel Bayzigitov

**Affiliations:** 1 R&D, Laboratory for Development Biotechnological Processes, R-Pharm JSC, Moscow, Russia; 2 Medical Department, R-Pharm JSC, Moscow, Russia; 3 BioPharmaceuticals R&D, R-Pharm JSC, Moscow, Russia

**Keywords:** artificial intelligence, biotechnology, complementary determining regions (CDRs), deep learning, machine learning, neural network

## Abstract

Monoclonal antibody–based therapeutics have become essential tools for treating infectious, autoimmune, and malignant diseases due to their high specificity and efficacy. As their clinical and scientific relevance continues to expand, the need for faster, more accurate and cost-effective development strategies has grown. Traditional laboratory-based methods for antibody design and improving remain reliable but are time-consuming, labor-intensive, and limited by experimental constraints. These challenges have driven a shift toward the integration of computational methods as a complementary approach for antibody engineering. The current review provides a simplified overall explanation of recent advancements in artificial intelligence (AI)-driven in silico tools used to accelerate and enhance the process of antibody discovery and optimization. We have systematically analyzed literature from clinical and research databases and summarized obtained data into a comprehensible overview. We highlighted how AI models contribute to sequence design, epitope-paratope predictions, affinity optimization, structural prediction and developability assessment.

In conclusion, the most effective strategy for next-generation monoclonal antibody development relies on the integration of computational prediction and design tools followed by experimental validation. Combining AI-driven innovation with traditional laboratory methods represents a powerful and complementary approach for achieving accurate, efficient, and clinically relevant antibody therapeutics.

## 1 Introduction

Therapeutic monoclonal antibodies represent one of the most significant innovations in modern medicine. They have been successfully applied across various therapeutic areas, particularly in oncology and immunology (1). Owing to their exceptional specificity and affinity toward target antigens, prolonged half-life, and lower incidence of adverse effects compared to conventional small-molecule drugs, monoclonal antibodies have rapidly become a dominant class of therapeutics in the biopharmaceutical market, especially considering the growing challenge of multidrug resistance (2). For instance, monoclonal antibodies have shown considerable promise in combating viral infections such as SARS-CoV-2, HIV, malaria, influenza, and Ebola (3, 4). Moreover, these molecules can also serve as a delivery system for active agents or toxins directed at specific cells or tissues. For example, antibodies conjugated with radioactive isotopes have been used to selectively target and destroy tumor cells, demonstrating clinical success in treating Hodgkin’s and non-Hodgkin’s lymphoma (5, 6).

Since the recognition of the therapeutic potential of monoclonal antibodies, numerous antibody engineering techniques have been actively developed, advancing antibody-based therapeutics. The early processes of antibody discovery and development involved multiple complex and time-consuming steps. In contrast, modern approaches increasingly rely on artificial intelligence (AI) and computational tools to design antibodies with optimized efficacy, specificity, and manufacturability. The integration of AI into early drug discovery offers a major advantage; it significantly reduces the cost and complexity of antibody development, as optimization at the discovery stage is far more efficient than at the late formulation and production phase. Early computational models for antibody design were limited by scarce data and inadequate computational capacity. However, with rapid advancements in sequencing technologies and the accumulation of large-scale datasets of antibody structures, sequences, binding affinities, and functional properties, AI-driven platforms have revolutionized the design and synthesis of biotherapeutics, paving the way for faster and more precise antibody development (7–10). AI models are now capable of analyzing vast datasets to construct specific computational frameworks that accurately predict and optimize key antibody attributes (11, 12). The rapid progress and integration of computational methods into immunology have given rise to a new interdisciplinary field: immunoinformatics, which applies bioinformatics and AI-based tools to analyze, interpret, and model complex immunological data (13, 14).

**Figure 1:**
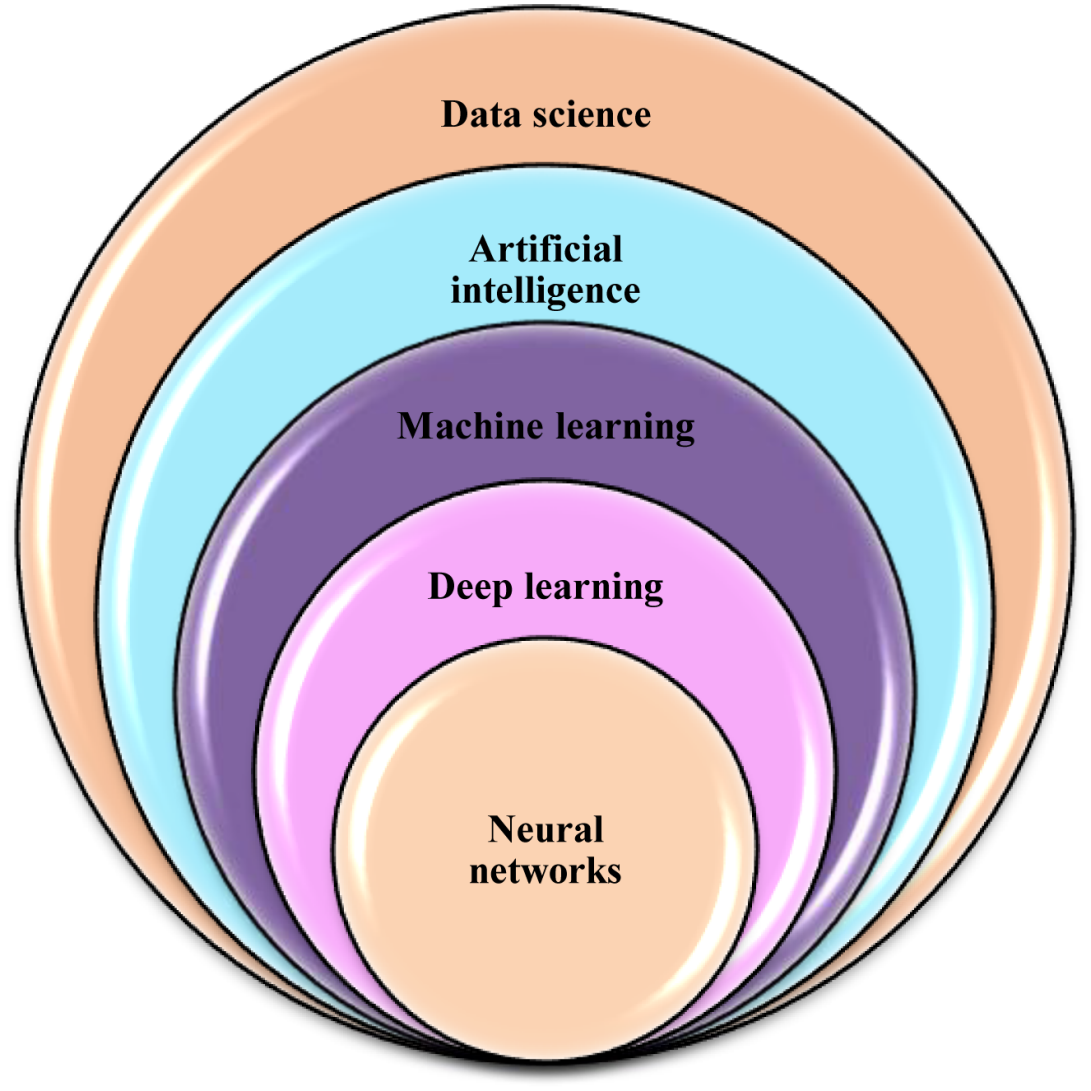
Conceptual hierarchy of data science. Artificial intelligence represents a subfield of data science and encompasses machine learning approaches. Machine learning includes deep learning and artificial neural networks models and techniques.

Artificial intelligence is at core dedicated to automating complex cognitive tasks typically performed by humans, enabling machines to execute these tasks with exceptional accuracy and speed. Within AI, machine learning (ML), artificial neural networks (ANNs), and deep learning (DL) represent key approaches and algorithms that drive intelligent behavior, **Figure 1** (15). ML, a major subfield of AI, allows computers to identify patterns and build predictive models from data rather than relying on explicit programming (16). In ML, the model is trained using input data and corresponding outputs to infer underlying rules and relationships, which can later be applied to predict outcomes for new datasets (17, 18). Traditional ML approaches often require manual feature selection by human experts. There are four principal types of machine learning (1); the supervised learning where the model learns from labeled datasets containing both inputs and known outputs (2); unsupervised learning in which the model analyzes unlabeled data to identify patterns, similarities, or groupings (3); reinforcement learning where the model learns through feedback from its environment, improving performance via trial and error and (4) semi-supervised or hybrid learning which combines labeled and unlabeled data to enhance model accuracy and robustness (15, 19).

Artificial Neural Networks (ANNs) represent another core branch of artificial intelligence. These models consist of multiple interconnected layers capable of automatically learning hierarchical data representations, analogous to how neurons in the human brain process information. Learning in ANNs occurs through iterative adjustment of connection weights to minimize prediction errors, typically using backpropagation algorithms. This optimization process is repeated many times until the model achieves optimal accuracy and predictive performance (20). The learning process begins at the input layer, which receives features such as numerical or categorical data. Each hidden layer (comprising artificial neurons) computes a weighted sum of its inputs, adds a bias term, and applies an activation function to introduce nonlinearity. The output layer then generates predictions. A loss function quantifies the difference between predicted and actual values, while the backpropagation algorithm calculates error gradients and updates the network’s weights using gradient descent to minimize the loss. Training iterations continue until the model converges and can be effectively applied to unseen data (15). There are several types of ANNs (1); feedforward neural network (FNN), the simplest form, where data flows unidirectionally from input to output (2), convolutional neural network (CNN) for image analysis using convolutional filters to detect spatial features and (3) recurrent neural network (RNN) for sequential data, this type considers temporal relationships between inputs (21). When neural networks include many hidden layers, they are referred to as Deep Neural Networks (DNNs), the foundation of deep learning. DL models can automatically extract features from raw, unstructured data (images, text, audio) and are particularly powerful for analyzing large and complex datasets such as medical imaging, speech, and video (22–24).

With the advancement of next-generation antibody technologies and the continuous evolution of methods for designing and optimizing biotherapeutics, a new generation of engineered antibodies has emerged (25, 26). These advanced antibodies offer enhanced specificity, accuracy, and therapeutic efficiency, allowing precise targeting of disease-related molecules. Traditional recombinant full-length monoclonal antibodies, although effective, often face limitations due to their large molecular size, which can hinder tissue penetration and complicate manufacturing. To overcome these challenges, smaller engineered antibodies known as single-chain variable fragments (scFvs) were developed. These molecules consist of the variable regions of the heavy and light antibody chains connected by a short, flexible polypeptide linker (typically 15–20 amino acids). scFvs are commonly expressed in E. coli systems, allowing efficient and cost-effective production. Owing to their compact size, they demonstrate superior tissue penetration and rapid clearance while eliciting minimal immune response, as they lack the constant (Fc) region. However, scFvs also have drawbacks, including a short half-life and a tendency to self-associate or aggregate under certain conditions (26–28). Another major innovation is the development of bispecific antibodies (bsAbs), which can simultaneously recognize and bind two distinct epitopes. This dual specificity enhances therapeutic potency and enables complex mechanisms of action. bsAbs can be generated using either traditional hybridoma fusion techniques or through genetic engineering approaches that construct synthetic genes encoding the desired bispecific format (29, 30). Furthermore, advances in antibody engineering have led to the emergence of multi-specific antibodies, capable of interacting with more than two targets simultaneously. These next-generation molecules represent a significant step forward in antibody therapeutics, offering greater efficacy, versatility, and potential for treating complex diseases (31).

Additionally, nanobodies (Nbs) are single-domain antibodies characterized by their small size (~15 kDa) and stability. Derived from camelid heavy-chain antibodies, nanobodies consist of a single variable domain (VHH) from the heavy chain. They can be efficiently produced in bacterial expression systems, making them cost-effective to manufacture. Nanobodies exhibit excellent tissue penetration, high affinity, solubility, and thermal stability, which make them valuable for both diagnostic and therapeutic applications. However, they also present certain limitations, including a shorter half-life, lack of effector functions due to the absence of the Fc region, and potential immunogenicity in humans (32, 33). Furthermore, Fc-engineered antibodies represent another class of advanced monoclonal antibodies that have been genetically or chemically modified in their Fc (constant region) to improve therapeutic performance. These modifications aim to reduce immunogenicity, extend serum half-life, or enhance effector functions. Engineering can be achieved through amino acid substitutions, targeted mutations, or alterations in post-translational modifications, particularly glycosylation profiles. It is crucial, however, that these modifications do not compromise antibody stability or developability-related properties, ensuring that the resulting molecules remain effective and manufacturable (34, 35).

Antibody–Drug Conjugates (ADCs) are hybrid therapeutic molecules that combine a monoclonal antibody designed against a specific tumor target with a cytotoxic small-molecule substance (payload). ADCs are designed to deliver chemotherapy directly to cancer cells, minimizing harm to healthy tissues; hence, they merge mAb precision with chemotherapy strength, avoiding systemic toxicity and enhancing drug efficacy. However, their development remains complex and costly, with challenges such as resistance and off-target toxicity (36, 37). Other next-generation therapeutic antibodies include antibody mimetics, engineered small peptide molecules that imitate the antigen-binding function of an antibody while lacking the Fc region, and antibody biosimilars, which are cost-effective, highly similar versions of approved monoclonal antibodies designed to provide the same therapeutic benefits while ensuring safety and efficacy despite minor production-related differences (26, 38).

In this review, we conducted a systematic literature search across several clinical and scientific databases, including PubMed, Scopus, Web of Science, and Google Scholar, analyzed data from studies about the advancements of artificial intelligence tools in biotechnology and the development and synthesis of monoclonal antibodies. Our objective is to provide non-technical biologists readers with a clear and accessible overview of AI-based instruments and models, explaining their core principles and data-processing mechanisms. Furthermore, we aim to present the most recent computational approaches currently applied in the design of biotherapeutics, highlighting their growing relevance in modern biomedical research.

## 2 Traditional biotechnological methods

Traditional biotechnological methods have laid the foundation for therapeutic proteins design, synthesis, and optimization or therapeutic, diagnostic, and research purposes, prior to the revolutionary advances in artificial intelligence–based modeling. Despite the growing reliance on in silico methods for protein engineering and development, traditional experimental techniques remain indispensable for validating computational predictions and ensuring the functional relevance of designed antibodies *in vitro* and *in vivo*. On the other hand, such methods are time-consuming, costly and labor-intensive, which limits their scalability and efficiency. Below, the main traditional methods used in antibody development are described.

### 2.1 Hybridoma

Hybridoma technology was first discovered and developed by Köhler and Milstein in 1975 (39). This technique enables the creation of an immortal cell system capable of producing monoclonal antibodies against a specific antigen. At the time of its discovery, it represented a major revolution in immunology and remains one of the most widely used methods in research and therapeutic antibody development (40–43). In this process, an experimental animal (typically a mouse or rabbit) is immunized with the antigen of interest to elicit an immune response and generate antigen-specific B cells. The antibody-producing B lymphocytes are then isolated and fused with immortal HAT-sensitive myeloma cells using polyethylene glycol–mediated fusion or electrofusion to form hybridoma cells. These hybridomas are subsequently selected in HAT medium, and the appropriate clones are identified by ELISA screening for specificity toward the target epitope. Once the desired hybridoma clone is selected, it is cultured *in vitro* or *in vivo* to produce large quantities of the monoclonal antibody (41).

### 2.2 B-cell immortalization technology

Immortalization of human B cells method preserves the essential characteristics and antibody-producing capacity of B cells *in vitro*. Immortalization can be achieved through cellular transformation using Epstein–Barr virus (EBV, 44, 45), or Simian virus 40 (SV40) infection (44), CD40 activation (45, 46), or other genetic and cellular engineering techniques (47), **Table 1**. The resulting immortalized cells acquire the ability to bypass senescence and proliferate continuously, allowing sustained monoclonal antibody production. This approach enables efficient generation of antibodies, including those that are rare or highly specific, and offers a key advantage, since the antibodies are of human origin, they exhibit reduced immunogenicity and improved compatibility for therapeutic use (48–52).

**Table 1:** Human B cell immortalization methods.

Method	Principle	Advantages	Limitations
Epstein–Barr virus (EBV) mediated B cell transformation	Latent genes of EBV activate cellular growth factor receptors and related signaling pathways enabling continuous cell division	Produces fully human antibodies, preserves native pairing	Narrow host specificity, genetic instability that may cause variable antibody secretion
Simian Virus 40 (SV40) - B-cell infection	LT antigens of SV40 trigger cellular transformation and enable sustained proliferation by distrusting the cell cycle checkpoints, and p53 function	This virus can infect a wide range of rodent and mammalian B cells, provides efficient immortalization of cells	Genetic instability, altered cell physiology
CD40-activation of B lymphocytes	B lymphocytes CD40-activation upon binding to its ligand CD40L, inhibits the programmed cell death and promotes cell proliferation, immunoglobulin class switching and cell surface markers expression, by triggering NF-KB, JNK, and p38 MAPK cellular pathways	Enabling antibody class switching, support large-scale production, good yield purity:	Complexity, efficiency depends on the nutrients and signaling components in the culturing environment
B cell gene editing	Activation of proto-oncogene Bcl-6 in B lymphocytes promoting survival and proliferation. prevent apoptosis and sustain cell growth by suppressing the expression of the tumor suppressor gene p53. Suppress both either p53-dependent or p53-independent pathways of growth arrest and apoptosis	Controlled editing, native pairing of human antibodies	Complex and labor

Immortalized human B cells represent a promising platform for producing diverse, naturally derived low-immunogenicity human antibodies suitable for a wide range of applications. They also serve as a valuable source for generating monoclonal antibody libraries. However, the current evaluation of their potential for large-scale antibody production remains limited (52).

### 2.3 Display technology

The concept of displaying specific peptides on the surface of bacteriophages was first introduced by George P. Smith in 1985 (53). This technique has since been refined and optimized for numerous research and clinical applications. The phage display system offers several advantages, including the ability to generate large libraries of diverse proteins with distinct properties and even rare variants, the capacity for selecting proteins with desired characteristics, and the retrieval of the corresponding genetic information (54–56).

Among various systems, Escherichia coli filamentous bacteriophages (Ff) are the most widely used for antibody display (57, 58). The coat proteins of filamentous phages interact with the F pilus on the surface of E. coli cells, facilitating the infection (59). In particular, the M13 filamentous bacteriophage is the most employed display system. It is a flexible, cylindrical virus with a circular single-stranded DNA genome containing nine genes that encode five coat proteins (pIII to pIX) and six assembly and replication proteins (60). Most phage display systems rely on pIII–antibody fusion proteins, as the pIII coat protein offers structural flexibility and can display relatively large proteins without compromising their functionality (61, 62). Co-infecting E. coli cells harboring a phagemid with a helper phage is a more efficient strategy for producing fully functional phage particles that display pIII–antibody fusions. This co-infection is necessary because the phagemid alone lacks the complete set of genes required to form a mature bacteriophage. The helper phage complements this deficiency by supplying the wild-type coat protein genes necessary for phage assembly and replication (63, 64).

Once the desired antibody gene is inserted into the phage genome, and this system has infected the host cell, large-scale production can begin. Clone selection is then carried out using purified, specific antigens immobilized on solid supports in a process known as biopanning (65). After several rounds of biopanning, ELISA is typically used to assess antigen-specific enrichment within the phage pools. The most enriched pools are screened to isolate high-affinity individual clones, which are then characterized through molecular analyses to identify unique antibody sequences and define their CDR regions (66). Once positive clones are identified, downstream processing depends on the intended application. Additionally, affinity maturation steps can be performed using mutant phages (67, 68).

Besides phage display, other display technologies such as yeast display (69), and cell-free ribosome display (70) have been developed. However, phage display remains the most widely utilized system due to its ability to generate highly diverse libraries and its relative simplicity and robustness.

The core principle of ribosomal display technology lies in the formation of a ternary complex consisting of a polypeptide (antibody), a ribosome, and its encoding mRNA, thereby maintaining a direct, non-covalent link between the genotype and the expressed phenotype. The general workflow involves constructing a diverse DNA library, followed by *in vitro* transcription and translation, and selecting ribosome–mRNA–antibody complexes that specifically bind to the target antigen. The removal of translational stop codons from the mRNA prevents the nascent peptide and its mRNA from being released from the ribosome, which stabilizes the complex (71, 72). The associated mRNA is then recovered, reverse-transcribed into cDNA via RT-PCR, and amplified to serve as a template for subsequent evolutionary and affinity maturation cycles, allowing the generation of antibodies with enhanced affinity and specificity (73, 74). Compared to cell-based display systems, ribosome display offers several key advantages: it can accommodate extremely large library sizes (up to 10¹^4^ variants), enables rapid evolution and supports the expression of toxic or unstable proteins that cannot be produced in living cells. Furthermore, the mRNA–protein linkage facilitates the simultaneous recovery of both the desired antibody and its genetic information. However, the system also has limitations, primarily related to mRNA instability and complex fragility (70).

### 2.4 Recombinant monoclonal antibody technology

Through the application of genetic engineering techniques, recombinant monoclonal antibodies can be efficiently synthesized. This approach enables precise editing and design of antibody structure and antigen specificity. The process begins with the isolation of the genes encoding the heavy and light chains of an antibody from B lymphocytes of an immunized organism. These genes are then cloned into suitable expression vectors containing a coding sequence of constant region, a strong promoter, and a selectable marker gene. The resulting recombinant vector is subsequently used to transform mammalian host cells, such as Chinese Hamster Ovary (CHO) or HEK293 cells, to produce the desired antibody (75, 76). Following transfection, clones are screened for antigen specificity, and the most promising ones are cultured and expanded for large-scale production. Recombinant antibody technology provides high consistency, scalability, and design flexibility, allowing the generation of chimeric, humanized, or fully human antibodies with reduced immunogenicity and enhanced therapeutic efficacy (77).

### 2.5 Transgenic animals

Engineering of animals’ genome for producing antibodies is a valuable tool used for both therapeutic and diagnostic purposes. Through immunization using antigen of interest, transgenic animals such as rodents and cattle can generate human-like monoclonal antibodies, which are subsequently isolated from their serum. To achieve this, advanced genetic engineering and genome-editing techniques are employed to introduce human immunoglobulin (Ig) gene loci into the animal genome. In rodents, large fragments of human Ig genes carried on bacterial artificial chromosomes (BACs) or yeast artificial chromosomes (YACs) can be inserted into oocytes via microinjection or introduced into embryonic stem (ES) cells through transfection. Similarly, in ruminants, somatic cell nuclear transfer (SCNT) from fibroblasts engineered to carry human chromosome fragments or human artificial chromosomes (HACs) enables the generation of transgenic lines capable of human antibody expression. For efficient antibody production, an inactivation of the animal’s endogenous Ig genes is essential to prevent competition between native and human loci and to enhance the yield of fully human antibodies. However, early models showed lower expression efficiency of human antibodies compared with endogenous ones, primarily due to suboptimal interactions between human constant regions and the host’s B-cell signaling components. This limitation was later overcome by linking human variable-region genes (V–D–J segments) to the host constant regions simultaneously with knocking out endogenous Ig genes, resulting in improved expression levels and robust immune responses (78). One of the major technical challenges in developing transgenic antibody models has been the large size and complexity of human Ig loci. Early strategies utilized smaller constructs of V–D–J segments, which, despite limited diversity, were able to produce functional monoclonal antibodies with satisfactory antigen-binding properties. With advances in molecular cloning, researchers successfully integrated larger and more complete Ig loci using plasmids, cosmids, BACs, and YACs. These innovations ultimately enabled the creation of transgenic rodent lines with chimeric immunoglobulin loci capable of mounting immune responses comparable to wild-type animals and generating antibody repertoires that closely resemble the diversity of the human immune system (79, 80).

## 3 AI tools in design and development of monoclonal antibodies

Computational and artificial intelligence (AI) models applying machine learning and deep learning algorithms have been developed to produce accurate predicting models based on interpretation, analysis and processing of data from large datasets (81). Hence, major roles in the development of AI models are played by these databases that contain huge and diverse information, including DNA and RNA sequences, amino acid sequences, three-dimensional (3D) structures of antibodies, epitopes and paratopes, as well as antibody–antigen complexes binding and interaction. Sequence databases are typically constructed by collecting and interpreting large-scale data obtained from sequencing technologies or protein analysis using mass spectrometry. Structural databases are generated using the results from empirical structural biology methods such as X-ray crystallography, electron microscopy, nuclear magnetic resonance (NMR) spectroscopy, and other techniques (82–84). Among several structural approaches, X-ray crystallography remains one of the most widely used methods for determining protein structure. In this technique, the 3D structure of a protein is resolved by processing and analyzing the diffraction pattern produced when an X-ray beam passes through a crystal of a highly purified protein at high concentration (85, 86). Analysis of the obtained diffraction data provides detailed information about crystal symmetry, molecular structure, atomic positions, unit cell dimensions, and electron density distribution (86, 87). X-ray crystallography can provide high-resolution protein structures information, enabling rapid characterization of molecular fragments and their physicochemical properties, which is highly relevant for molecular engineering. This is particularly important in the development of biotherapeutics and antibody design. Obtained structural information from these methods are essential for identifying active sites and binding regions of proteins. Furthermore, determining the 3D structure helps elucidate molecular behavior, mechanisms of action, and potential interactions with other molecules (88, 89). However, this technique faces several challenges when applied to antibodies. These include the difficulty of obtaining high-quality crystals of antibodies or antibody–antigen complexes at sufficiently high concentrations. The intrinsic conformational flexibility of antibodies further complicates structural analysis. Additionally, the relatively large molecular size of antibodies can hinder the crystallization process (81, 82).

Both structural and sequence datasets represent important resources for training machine and deep learning models to improve AI-based tools and enhance their predictive performance in antibody engineering and development, which could ultimately accelerate development timelines, costs and efforts. AI-driven approaches are increasingly employed in several stages of antibody design and development like epitope selection, paratope and CDR structure prediction and optimization, protein structure modeling and discovery of potential antigen-binding sites (90). Moreover, computational models assist in evaluating and improving the developability of the candidate antibodies. In the following section, we provide an overview of recent AI-based computational tools used for antibody design and enhancement (7).

Developed AI-based approaches significantly accelerate development timelines along with reducing the cost and effort required to design new biotherapeutic drugs or other bio-functional proteins. AI-driven approaches are increasingly employed in several stages of antibody design and development like epitope selection, paratope and CDR structure prediction and optimization, protein structure modeling and discovery of potential antigen-binding sites. Moreover, computational models assist in evaluating and improving the developability of the candidate antibodies. In the following section, we provide an overview of recent AI-based computational tools used for antibody design and enhancement (90).

### 3.1 Epitope prediction

An antibody recognizes and binds its target through a specific site on the antigen known as the epitope. Information about the binding epitope can guide the entire development process of a candidate antibody (91). Epitopes functionally can be divided into B-cell epitopes and T-cell epitopes, both of which induce different types of immune responses upon binding to their respective targets. From a structural perspective, epitopes can be classified as linear or conformational. Linear epitopes consist of continuous amino acid residues, whereas conformational epitopes are formed from sequence-wise discontinuous amino acids that are spatially adjacent (92). Several computational approaches are employed to map epitopes, **Table 2** (93). Such in Silico methods assist in identifying antigenic regions that can be targeted by antibodies, playing a pivotal role in designing therapeutic antibodies with high specificity and minimal off-target effects (94, 95).

**Table 2:** AI-based tools and computational approaches for epitope prediction.

AI tool	Principle	Site	Advantages	Limitations
BepiPred-3.0	sequence-based linear epitope predictor, DL^1^, PLM^2^	https://services.healthtech.dtu.dk/services/BepiPred-3.0/	leverages large-scale datasets, precision	Dependent on pretrained model quality; computationally intensive
EPMLR	sequence-based linear epitope predictor, multiple linear regression with 10-fold cross-validation	–	Interpretable statistical model; moderate sensitivity and AUC	limited ability to model complex patterns
EpiPred	structure-based conformational epitope predictor, ML^3^	https://web.mit.edu/stern/www/epipredmain.html	Specific epitope prediction for the applied antibody, suitable for discontinuous epitopes	Requires available clear 3D structure databases
SEPPA 3.0	structure-based conformational epitope predictor, Logistic Regression	–	Applicable to glycoproteins; improved datasets; good AUC and balanced accuracy	Performance still limited compared to experimental mapping
Epitope3D	structure-based conformational epitope predictor, ML	–	Trained on updated datasets for spatial epitope prediction	Affected by structural data availability
DiscoTope 3.0	hybrid models. ML combined with cross-validation testing	https://services.healthtech.dtu.dk/services/DiscoTope-3.0/	Scalable, integrated with AlphaFoldDB & RCSB, provides accurate predictions of spatial epitope	Requires high-quality structures datasets
EM-DMS	hybrid models, DL	–	High-resolution mapping; captures functional binding effects, rapid mutation effect analysis	Experimental cost and complexity
SEMA	hybrid models, DL	https://sema.airi.net/	Captures complex structural relationships; ranks immunodominant regions	Requires extensive training datasets

BepiPred is an AI model for linear epitope mapping and analyzing contiguous amino acid sequences. It predicts linear epitopes by combining a hidden Markov model with one of the most effective propensity scale approaches (96). BepiPred still has limitations and requires further refinement to provide reliable predictions of B-cell epitopes. Consequently, the next-generation web server, BepiPred-2.0, was developed. This sequence-based B-cell epitope prediction tool was trained on datasets derived from epitope crystal structures, offering higher quality data and improved predictive performance (97). A more advanced version, BepiPred-3.0, further enhances prediction accuracy by leveraging protein language models (LMs) trained on large datasets of protein sequences and structures, allowing it to predict epitopes directly from antigen sequences with superior precision (98).

Another sequence-based epitope predictor is the EPMLR model for linear B-cell epitope prediction based on antigen sequence. It utilizes multiple linear regression with a ten-fold cross-validation test, achieving 81.8% sensitivity, 64.1% precision, and an area under the receiver operating characteristic curve (AUC) of 0.728 (99, 100).

As more antibody–antigen structural complexes have been added to the Protein Data Bank (PDB, 102), several computational approaches utilizing structure-based information have been developed to predict conformational B-cell epitopes too. Among these are EpiPred (101) and the web server BepiPred-2.0, which employs a random forest algorithm trained on epitopes annotated from antibody–antigen protein structures (97).

An advanced version of the logistic regression model, the Spatial Epitope Prediction of Protein Antigens (SEPPA 3.0), enables epitope prediction in glycoproteins with an AUC of 0.749 and a balanced accuracy of 0.665. The parameters in SEPPA 3.0 were updated based on the rich available datasets (102). Similarly, Epitope3D, trained on the latest datasets, provides predictions of spatial epitopes using a machine learning algorithm combined with cross-validation testing, outperforming earlier available approaches in epitope prediction (103). Although these methods have demonstrated improved performance compared to random prediction procedures, a comparative study by Cia et al. reported that their ability to accurately predict conformational epitopes remains limited, indicating the need for further optimization and performance enhancement (104).

There are also hybrid models that predict both linear and conformational epitopes across multiple independent datasets, such as DiscoTope. The latest version, DiscoTope-3.0, employs inverse folding structure representations combined with a positive–unlabeled learning strategy, enabling the analysis of over 100 protein structures per submission. Moreover, the integrated server interfaces with both research collaborator for structural bioinformatics protein data bank (RCSB PDB) and AlphaFoldDB, allowing large-scale epitope prediction across more than 200 million cataloged proteins (105). Additionally, the web-based tool ElliPro (106) utilizes both sequential and structural information to enhance the accuracy and visualization of predicted epitopes. Beyond epitope prediction, ElliPro can also be applied to characterize clinically relevant epitope repertoires in HLA matching, providing valuable insights for transplantation processes (107, 108). Moreover, an advanced epitope mapping technique employ deep mutational scanning (DMS) to systematically analyze all possible amino acid substitutions by mutations, thereby providing valuable insights into the structural characteristics of both linear and conformational epitopes (109). For instance, epitope mapping–DMS (EM-DMS) can rapidly generate information on how individual non-synonymous mutations affect antigen–antibody binding and interactions. This approach plays a crucial role in the development of therapeutic antibodies. During the COVID-19 pandemic, EM-DMS played an essential role, where rapid assessment of mutational impacts on antibody recognition was essential.

The SEMA (Spatial Epitope Modelling with Artificial intelligence) model predicts discontinuous B-cell epitopes by integrating information from both the primary amino acid sequence and the tertiary spatial structure of the antigen. Developed through a transfer learning framework, SEMA leverages pretrained deep learning models trained on extensive datasets of antigen–antibody complexes. This approach enables the model to capture intricate structural relationships that define epitope formation. Notably, the authors demonstrated that SEMA can quantitatively identify and rank immunodominant regions within the receptor-binding domain (RBD) of SARS-CoV-2, highlighting its potential for precise epitope characterization in viral antigen research (110, 111).

Epitope prediction faces several challenges, including variations in antigen origin, flexible conformational changes, epitope accessibility, and limited availability of high-quality datasets for machine training (94, 112). To address these limitations, it is essential to integrate experimental validation with the refinement of computational algorithms and the expansion of diverse and comprehensive training datasets. Such combined efforts can significantly improve the accuracy and reliability of epitope prediction models. More advanced ML-based tools, such as EpitopeVec, have demonstrated improved performance by leveraging large-scale databases and incorporating amino acid residue properties together with protein language models and antigenicity scales. This approach enhances cross-testing accuracy and enables more precise prediction of linear epitopes (113). 

For T-cell epitope prediction, computational tools are also increasingly applied in both clinical research and therapeutic development. One example is TepiTool, an integral part of the Immune Epitope Database (IEDB), which provides predictive analysis of epitopes across multiple species and supports a wide range of immunological applications (114, 115).

### 3.2 CDRs optimization and paratope prediction

**Figure 2:**
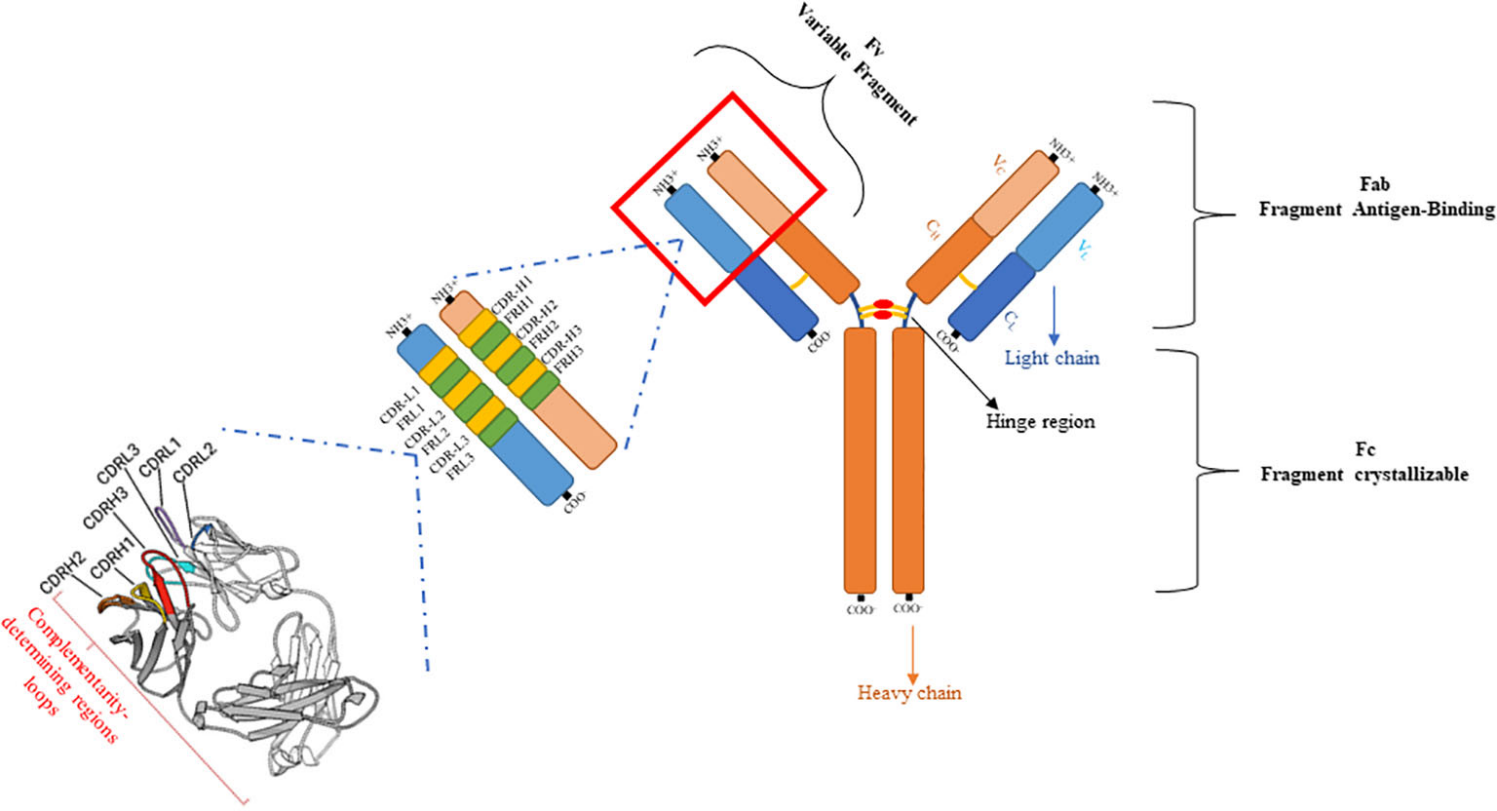
Antibody structure; antibody consists of two antigen-binding fragments (Fab) and crystallizable fragment (Fc). Each Fab arm contains variable and constant fragments from both heavy and light chains (V_H_, C_H_, V_L_, C_L_). The variable fragment of Fab comprised of framework domains and CDR loops formed from both heavy and light chains.

An antibody is mainly composed of two regions: the variable (Fab) region and the constant (Fc) region. The variable fragment (Fv) of the antibody consists of two key components; the framework (FR) and the complementarity-determining regions (CDRs). The framework itself is divided into the light-chain framework (FRL) and the heavy-chain framework (FRH). Similarly, the CDRs are composed of light-chain CDRs (L) and heavy-chain CDRs (H). The CDRs form three loops within the variable region of each chain, designated as CDR-L1 to CDR-L3 in the light chain and CDR-H1 to CDR-H3 in the heavy chain, **Figure 2** (116). Among these six loops, the CDR-H3 region is the most variable and plays the most critical role in antigen binding (117).

CDR sequences define the conformational loops responsible for paratope formation and antigen binding. Optimization of the CDR sequences is a crucial step in enhancing an antibody’s binding affinity and specificity toward its target, also in reducing immunogenicity, thereby improving the overall efficacy and safety of the therapeutic candidate. AI methods are widely used to predict and optimize this region, **Table 3**. For instance, DeepH3, and its more advanced successor DeepAb, are deep learning–based approaches that predict the CDR-H3 loop structure using geometric potentials, inter-residue distance maps, and orientation data, in a manner conceptually similar to TrRosetta and the original version of AlphaFold (117–119).

**Table 3:** AI-based tools and computational approaches for CDR optimization.

AI tool	Principle	Site	Advantages	Limitations
DeepH3	Loop predictor, DL	https://github.com/Graylab/deepH3-distances-orientations	Accurate CDR-H3 loop structure prediction	Focused on H3 loop
DeepAb	Loop predictor, DL	https://github.com/RosettaCommons/DeepAb	Improved accuracy, end-to-end antibody structure prediction, Extends DeepH3 to full antibody variable regions	Computationally intensive
ABlooper	Loop predictor, DL	https://github.com/oxpig/ABlooper/blob/master/ABlooper/ABlooper.py	Leverages equivariant graph neural network for rapid high accurate loop prediction	heavy model, computationally intensive
AbFlex	Loop predictor, DL	https://github.com/wsjeon92/AbFlex/blob/main/AbFlex.py	Targets loop flexibility as determinant of affinity, energy-based modeling of binding kinetics	requires large structural datasets
OptMAVEn 2.0	Immunogenicity improver, ML	https://github.com/maranasgroup/OptMAVEn_2.0/blob/master/OptMAVEn-2.0	Dual optimization: affinity + immunogenicity, *De novo* design of antibody variable regions	Computational complexity
EquiPocket	loop predictor, Immunogenicity improvement, DL	https://github.com/fengyuewuya/EquiPocket	Precisely predict loop structure using E (3)-equivariant geometric graphic neural network, Useful for epitope–paratope mapping	Requires reliable structural models
OptCDR	loop predictor, Immunogenicity improvement	https://www.maranasgroup.com/submission/OptCDR_2.htm	*De novo* CDR sequence generation with enhanced affinity binding, aware of the immunogenicity	Depends on structural assumptions
Ens-Grad	Loop predictor, ML	–	Use convolutional neural network (CNN) architecture, to design CDR sequences with defined specificity	Complex training pipeline
PALM-H3	Loop predictor, DL	–	Uses generative protein language model for high precision CDR-H3 generation	Task-specific (H3-focused)
AbGAN-LMG	Loop predictor, DL	https://github.com/Zhaowenbin98/AbGAN-LMG	Integrates a language model with a generative adversarial network (GAN) framework to design and predict high-quality candidate antibody CDR sequences	Training instability typical of GANs
IgDiff	Loop predictor, DL	https://github.com/amelie-iska/IgDiff	Uses SE (3)-equivariant diffusion model to design variable domains with developability focus	High computational cost
IgLM	Generative modeling, DL	https://github.com/Graylab/IgLM	This model supports full-length antibody design, producing diverse CDR loop libraries with enhanced in silico developability profiles and reduced immunogenicity risk, multi-species	Requires massive training data
Hu-mAb	Humanness classifier, ML	https://opig.stats.ox.ac.uk/webapps/sabdab-sabpred/sabpred/humab	Reduces immunogenicity; suggests substitutions	Focused primarily on human-likeness
SCALOP	canonical loop classifier, ML	–	Fast; high accuracy for non-H3 loops, Suitable for large-scale repertoire analysis	Does not model H3

Another deep learning-guided CDR loop predictor is ABlooper, which achieves high-speed and accurate modeling of antibody CDR conformations using equivariant graph neural networks. The method is a trade-off between speed and accuracy, with accuracy that is comparable to more computationally expensive structure prediction models (117). There is also the AbFlex, which was designed to accurately capture CDR loop flexibility as the key determinant of antibody–antigen binding kinetics. Trained on a large dataset of antibody crystal structures, AbFlex combines structural variation and energy considerations, and this enhances its predictive potential for flexible loop conformation (120).

Traditionally, antibody affinity maturation is achieved by generating large libraries of antibody variants through techniques such as error-prone PCR, random mutagenesis, or site-directed mutagenesis. These methods enable the creation of numerous antibody variants that can subsequently be screened for enhanced target-binding affinity. However, despite their utility, such approaches are labor-intensive, time-consuming, and unpredictable (121). To overcome these limitations, it is more time- and cost-efficient to employ computational in silico approaches for binding affinity prediction and improvement. These methods can estimate the potential impact of specific amino acid substitutions on antibody–antigen binding and overall interaction energy, providing a rational foundation for further experimental validation. Typically, such computational strategies rely on three-dimensional structural models of antibody–antigen complexes or, when unavailable, on protein sequence data and inferred structural features (122).

The potential immunogenicity of a monoclonal antibody refers to its ability to trigger an undesired immune response upon clinical administration. Immunogenicity remains one of the most significant challenges in the development and clinical application of therapeutic antibodies. Therefore, performing a thorough immunogenicity risk assessment prior to clinical trials is of critical importance, as it ensures the development of safe and effective biotherapeutics. Such assessments not only help identify potential immune-reactive regions within the antibody sequence but also inform key clinical decisions regarding dosage, administration route, and treatment duration, thereby ultimately enhancing therapeutic efficacy (123). Traditionally, antibody humanization has been performed through a largely empirical, trial-and-error approach. However, with advances in computational biology and artificial intelligence, numerous in silico algorithms and machine learning–based tools have emerged to support protein-engineering strategies. These approaches enable the optimization of amino acid sequences to reduce immunogenic potential, while simultaneously improving stability, binding affinity, and overall efficacy (124, 125). In this context, targeted amino acid substitutions, often introduced through rational mutagenesis, are increasingly being guided by machine learning and deep learning models. These models are capable of predicting beneficial mutations within the antibody sequence that can enhance binding affinity, reduce immunogenicity, and maintain structural integrity, offering a data-driven alternative to traditional experimental screening and accelerating the antibody optimization process (126–129).

Several computational frameworks have been developed for the *de novo* design of antibody variable regions with improved affinity and specificity toward a given target antigen. One such approach is OptMAVEn (Optimal Method for Antibody Variable Region Engineering) and its improved version, OptMAVEn-2.0, which are capable of capturing critical structural and energetic features that govern binding affinity (130–132). Notably, OptMAVEn also incorporates immunogenicity reduction procedures, allowing for the generation of multiple computationally designed antibody candidates that combine enhanced binding affinity with lower predicted immunogenicity. The dual optimization approach makes OptMAVEn a powerful tool for affinity maturation and humanization of therapeutic antibodies (131).

Another recent development, EquiPocket, employs an E (3)-equivariant geometric graph neural network to predict protein binding sites with good precision. This model integrates three computational modules to extract geometric and physicochemical information from both amino acid sequences and conformational structures, thereby enabling accurate modeling of potential binding pocket architectures within target proteins. Such methods are instrumental in rational antibody design, guiding both epitope–paratope mapping and affinity optimization in silico (133).

Optimal CDR (OptCDR) is an in silico computational framework designed to predict and generate optimal amino acid sequences for the antibody binding site. This approach enables the *de novo* design of antibodies with enhanced binding affinity and specificity toward their target antigens (132, 134). In addition to affinity optimization, OptCDR incorporates strategies to minimize immunogenic non-human residues within the designed CDRs, thereby improving structural compatibility with human antibody frameworks and enhancing their potential for therapeutic applications (9).

Another notable model, Ens-Grad, is aML–based method derived from a high-capacity convolutional neural network (CNN) architecture, capable of designing CDR sequences for human antibody candidates with precisely defined antigen-binding specificities (135, 136). By integrating ensemble learning with gradient-based optimization, Ens-Grad allows for efficient exploration of antibody sequence space, facilitating *de novo* antibody design.

A more recent advancement, the Pre-trained Antibody Generative Large Language Model (PALM-H3), focuses on *de novo* generation and prediction of the CDR-H3, which plays a key role in antigen recognition. PALM-H3 demonstrates good prediction accuracy and target-specific precision, successfully producing antibodies that exhibit strong and specific binding affinities against multiple SARS-CoV-2 variants (136). Furthermore, the same research group developed A2Binder, an AI-based model designed to predict and evaluate the binding specificity and affinity between antigen epitopes and antibody paratopes, further advancing computational antibody design and affinity maturation (136).

The Language-Model-Guided Generative Adversarial Network (AbGAN-LMG) integrates a language model with a generative adversarial network (GAN) framework to design and predict candidate antibody sequences. This model leverages the contextual understanding of language models to guide the GAN in producing structurally and functionally relevant antibody sequences. AbGAN-LMG–generated sequence libraries were evaluated for SARS-CoV-2 and Middle East Respiratory Syndrome (MERS-CoV), demonstrating that the model successfully learns the fundamental structural and physicochemical characteristics of antibodies while enhancing the diversity and quality of its generated sequence libraries (137).

IgDiff framework is a SE (3)- diffusion model that uses information of the amino acid sequence of an antibody to predict variable domains of candidates with good developability, designability and novelty (138). A related SE (3)-flow matching model, IgFlow, similarly enables *de novo* design of complete CDR loop structures with high structural self-consistency and fidelity (139).

Another advanced deep learning framework is the Immunoglobulin Language Model (IgLM), that integrates deep generative modeling (DGM) principles. Trained on an extensive dataset of 558 million antibody sequences, IgLM employs a text-infilling strategy with bidirectional contextual learning, allowing it to generate variable-length antibody sequences across multiple species. This model supports full-length antibody design, producing diverse CDR loop libraries with enhanced in silico developability profiles and reduced immunogenicity risk (140).

For reduced immunogenicity there is Hu-mAb a computational tool designed to evaluate the humanness (or human-likeness) score of antibody variable regions. The model is built on a robust machine learning classifier trained on a large-scale antibody sequence database, enabling it to effectively distinguish human from non-human antibodies. In addition to classification, Hu-mAb can also suggest amino acid substitutions that minimize potential immunogenicity while maintaining antigen-binding efficacy (141). Moreover, BioPhi platform provides automated antibody design with customized developability and therapeutic properties. BioPhi integrates a deep learning model Sapiens, which performs in silico humanization of antibody candidates, with OASis, which evaluates their humanness. Sapiens is trained on the Observed Antibody Space (OAS) database using a language modeling approach, allowing it to capture antibody sequence patterns and evolutionary relationships. Meanwhile, OASis provides precise immunogenicity assessments, helping to identify sequences with reduced immune risk. The combined BioPhi framework generates computational predictions closely matching experimental *in vivo* results, demonstrating its reliability and practical utility in antibody engineering (142, 143).

Another computational tool designed for annotating and predicting canonical loop structures of non-H3 antibody CDRs is SCALOP. This sequence-based model utilizes an automatically updated database and analyzes input antibody sequences by assigning variable-region loops with ANARCI numbering before classifying each CDR loop into its corresponding canonical structure. SCALOP demonstrates high predictive accuracy while operating significantly rapid, making it particularly suitable for large-scale antibody repertoire analysis and design workflows (144). Additionally, Gradient Boosting Machine (GBM) is a machine learning algorithm that was developed to enhance CDR structural prediction by classifying non-H3 CDR loops of antibodies candidate into structural clusters based on their amino acid sequences, increasing accuracy of predicting loop conformations. By integrating features derived from sequence of the antibody and leveraging the predictive power, GBM can effectively capture complex sequence–structure relationships, providing valuable insights for antibody design and engineering (145).

In addition to CDR prediction and optimization, AI-driven tools have also developed for antibody paratope accurate prediction. For instance, Paragraph model focuses on paratope prediction from the 3D structure of the antibody as input. Through the use of geometric deep learning approaches, Paragraph can predict antigen-binding residues highly accurately, consistently outperforming state-of-the-art paratope prediction methods. Its potency was confirmed through large-scale training and validation on datasets of antibody–antigen complexes (146). On the other hand, ProtTrans, brings protein language model usage to antibody research. It predicts paratopes from amino acid sequences in an antigen-agnostic manner with pre-trained transformer-based models originally developed for generic protein representations. In doing so, ProtTrans is able to achieve good predictive accuracy even without antigen structural data, thereby serving as a generalizable tool for large-scale antibody discovery and engineering (147).

Existing computational tools follow several complementary methodological principles; sequence-based tools to infer loop structures or paratope residues, structure-based methods to predict the 3D conformation and flexibility of CDR loops and generative frameworks that suggest novel CDR sequences with improved specificity and immunogenicity. Modern approaches illustrate an important trend toward integrated design and enhancement strategies that simultaneously optimize affinity, stability, and immunogenicity risk preserving developability attributes. However, despite these advances, many current computational tools still face limitations, particularly in achieving accurate, context-specific prediction and automation of complementarity-determining region design and optimization. Continued improvements in model generalization, data diversity, and integration of structural information are necessary to enable fully automated generation of antibodies with optimal antigen-binding affinity, specificity, and developability.

### 3.3 Antibody-antigen interactions

Antibodies exert their biological function after a specific binding to their target antigens. The high variability within the variable regions of antibodies allows for highly specific recognition and interaction with the corresponding antigen, leading to the activation of immune mechanisms necessary to achieve therapeutic effect (148). This binding is mediated by non-covalent interactions, including hydrogen bonds, van der Waals forces, hydrophobic interactions, and ionic bonds, which together ensure a strong yet reversible attachment. The specificity and strength of antibody–antigen binding are largely determined by the conformational structures of the antibody paratope and the antigen epitope structures that arise from the precise folding of amino acid residues within the CDRs and epitope regions (149). Several computational tools and algorithms have been developed for in silico prediction of antibody–antigen interactions, **Table 4**. One notable example is AbAgIPA, a neural network model that integrates geometric and spatial representations of antibody–antigen complexes. Unlike traditional sequence-based approaches, AbAgIPA analyzes not only the amino acid sequences but also the antibody gene sequences, incorporating rotational invariance through a modified Invariant Point Attention (IPA) mechanism. This ensures that complex predictions remain consistent regardless of protein orientation or spatial alignment, resulting in more accurate and robust predictions of antibody–antigen interactions (150).

**Table 4:** AI-based tools and computational approaches for antigen-antibody interaction prediction.

AI tool	Principle	Site	Advantages	Limitations
AbAgIPA	DL	https://github.com/gmthu66/AbAgIPA	geometric & spatial modeling with modified Invariant Point Attention (IPA), Integrates antibody amino acid sequences *and* antibody gene sequences, robust geometric reasoning	Requires complex input representations
DLAB	DL	–	Provides large-scale antibody screening and docking refinement	Requires 3D structural models
AbAgIntPre	DL, using Siamese-like architecture;	http://www.zzdlab.com/AbAgIntPre/	Works without structural data; strong performance	Limited structural interpretability
PECAN	Graph convolutional network with transfer learning, DL	–	Incorporates physicochemical properties; good generalization	Model complexity; data-scarcity
RLEAAI	Convolutional neural network with protein language model, DL	–	High sensitivity; strong CDR interaction modeling	Computationally intensive

Additionally, the Deep Learning for Antibodies (DLAB) framework represents a structure-based approach for predicting antibody–antigen interactions. DLAB enables virtual screening of candidate antibodies against target antigens, even when no prior information about known binders is available, allowing for prediction of potential binding and interaction sites. Moreover, DLAB enhances antibody–antigen docking by improving pose ranking and facilitating the selection of complexes for which reliable docking poses can be correctly generated (151).

Additionally, AbAgIntPre is a deep learning–based model designed to predict antibody–antigen interactions using only the amino acid sequences of the antibody and antigen. It employs a Siamese-like convolutional neural network architecture and demonstrates robust predictive performance, achieving an AUC of 0.82. When applied to the SARS-CoV dataset, AbAgIntPre exhibited high accuracy in predicting interactions, underscoring its potential utility in antibody design and optimization. PECAN is another deep learning framework developed for predicting antibody–antigen interactions. It incorporates the physicochemical properties of local interaction regions from both the antibody and antigen, employing a graph convolutional network (GCN) architecture. PECAN utilizes transfer learning to leverage the large amount of available structural and sequence data, thereby improving its generalization and predictive performance (152). Another graph-based machine learning model, CSM-AB, focuses on predicting antibody–antigen interface regions by analyzing structural features and atomic interactions within both the epitope and paratope. CSM-AB demonstrates strong performance in both cross-validation and blind testing, showing effective binding affinity prediction and practical utility in re-scoring docked antibody–antigen complexes (153).

The RLEAAI model represents a more recent DL-based computational approach designed to enhance the accuracy of antibody–antigen complex prediction. It employs a K-spaced amino acid pairs strategy to extract detailed sequence-based features, which are combined with representations from a pre-trained protein language model. The framework integrates convolutional neural network (CNN) along with recurrent criss-cross attention mechanism to generate its predictions. RLEAAI achieved superior performance for both HIV and SARS-CoV-2 datasets, displaying high sensitivity, particularly in capturing interactions involving complementarity-determining regions (154). Collectively, these models contribute to the advancement of computational antibody engineering, improving the efficiency and precision of therapeutic antibody discovery (155).

Predicting antibody–antigen interactions remain challenging due to the dynamic and flexible nature of protein conformations, the diversity of epitope–paratope interfaces, and the limited availability of experimentally validated complex structures for model training. As a result, AI models may not fully capture the conformational flexibility that occurs during actual binding events. Future improvements will likely rely on integrating multi-scale data sources, including molecular dynamics simulations, high-throughput mutational scanning, and large-scale structural predictions generated by modern protein structure models. By combining such datasets with advanced AI architectures, the reliability of interaction predictions could be significantly enhanced, enabling more accurate in silico screening of therapeutic candidates prior to experimental validation.

### 3.4 Developability assessment

Developability is a critical factor in the design and development of biotherapeutic. It reflects the overall feasibility of advancing a candidate antibody toward clinical and industrial production, based on its conformational, biophysicochemical, and pharmacological properties. These characteristics collectively determine key therapeutic outcomes, including pharmacokinetics, efficacy, and antigen-binding specificity. Developability assessment typically relies on analyzing the amino acid composition, charge distribution, and folding behavior of the antibody to predict essential attributes such as manufacturability, immunogenicity, solubility, precipitation, specificity, stability, storability, hydrophobicity, electrostatic properties, aggregation propensity, and viscosity (156, 157). These developability-related parameters strongly influence formulation stability and storage conditions, ensuring both therapeutic efficacy and long-term viability of the product (158). Assessing developability at the early stages of antibody discovery is highly advantageous, as it helps identify and resolve potential issues before costly later development. Early protein engineering interventions can correct problematic features such as aggregation or low solubility, thereby preventing investment in non-viable candidates.

*De novo* antibody design is strongly influenced by both the amino acid sequence and three-dimensional structure, which directly affect stability, solubility, and manufacturability. Consequently, several comprehensive databases have been established to store information of antibody sequences, structural features, and physicochemical properties, providing the foundation for the development of modern computational prediction methods. These databases are particularly critical as they supply training datasets for machine learning and deep learning models, enabling accurate prediction and optimization of developability attributes. In recent years, advanced computational approaches have emerged that can rapidly predict developability-related parameters of antibody candidates, significantly accelerating early-stage screening and design, **Table 5** (90, 159).

**Table 5:** AI-based tools and computational approaches for developability assessment and improvement.

AI tool	Principle	Site	Advantages	Limitations
SOLpro	sequence based – solubility/precipitation predictor, ML (SVM)	–	Early-stage solubility prediction; mutation suggestions, enhance solubility in high-concentration formulation	Limited structural context
CamSol	sequence-based - Physicochemical modeling	–	Predicts solubility across pH; mutation suggestion, Strong experimental validation	Not DL-based
PaRSnIP	sequence-based solubility predictor, ML	–	High accuracy at high concentrations, developability screening	Model interpretability
PROSO II	sequence based - solubility predictor, ML	–	Trained on expanding experimental datasets	No direct structure modeling
solPredict	sequence-based, protein language model enhanced, DL	–	Good performance for high-concentration mAbs formulations	Statistical predictions
ESM1b	sequence-based, DL	–	Evaluates mutation effects on solubility & stability	validation requirements
DeepSol	sequence based – solubility/precipitation predictor, ML	https://zenodo.org/records/1162886	, predicts protein solubility directly from amino acid sequences using a convolutional neural network architecture	Focused on sequence only
Aggrescan3D	Structure-based aggregation scoring	https://biocomp.chem.uw.edu.pl/A3D2 /	Mutation suggestions; strong visualization	Requires 3D structure input
CABS-flex	Structure-based flexibility simulations	https://biocomp.chem.uw.edu.pl/CABSflex2	Captures conformational variability, aggregation analysis	Approximate dynamics
FoldX	Structure-based, energy-based	https://foldxsuite.crg.eu/about#consortium	Stability-preserving design, mutation suggestion	requires large structure-databases
High Viscosity Index (HVI)	sequence based - solubility predictor, ML	Computational tool	employs logistic regression and decision tree algorithms to perform Rapid viscosity screening	Limited to variable domain features

One notable high-throughput computational method for assessing antibody developability is the Therapeutic Antibody Profiler (TAP). TAP requires the amino acid sequences of the antibody’s variable domains as input. It then employs ABodyBuilder2 to construct a 3D structural model of the variable region and evaluates five key developability metrics, including properties such as hydrophobicity and aggregation propensity. These metrics are compared against reference guidelines derived from a large library of clinically developed antibodies, under the assumption that such antibodies possess favorable developability profiles. Moreover, TAP utilizes continuously updated datasets of clinical therapeutics to maintain accuracy and relevance in its evaluations (160, 161).

Additionally, XGBoost and PyCaret constitute a machine learning workflow that leverages the eXtreme Gradient Boosting (XGBoost) algorithm to efficiently predict developability features of antibody candidates based on their biophysical and structural properties. This workflow enables the accurate prediction and evaluation of attributes such as hydrophobicity patches, surface charge distribution, and CDR loop length. The PyCaret module is then applied for training, tuning, and validating the best-performing predictive models across a large dataset comprising approximately 250,000 antibody models (9, 158). Additionally, several structure-based computational approaches have been developed to predict and optimize crucial biophysical and pharmacokinetic properties of antibodies including isoelectric point (pI), viscosity, stability, surface charge, and clearance rate, all of which are vital for achieving optimal high-concentration formulations suitable for therapeutic use (162, 163).

Despite rapid progress in this area, the assessment and evaluation of developability attributes for biotherapeutics remain a significant challenge, largely due to the limited availability of large, high-quality experimental datasets. Expanding and curating such datasets is essential to further improve the accuracy and generalizability of machine learning and deep learning-based prediction models. The aforementioned tools and methods can be applied to predict the overall developability profile of a therapeutic antibody candidate. In parallel, several specialized computational tools have been developed to estimate individual developability-related attributes, focusing on specific physicochemical or structural properties of antibody candidates, as described below.

#### 3.4.1 Solubility and precipitation

Solubility and precipitation are critical factors influencing the developability and manufacturability during the development of a candidate antibody. Antibody precipitation can occur during cell cultivation, downstream processing, or formulation, potentially leading to reduced purification efficiency as well as compromised product yield and stability. Furthermore, insufficient solubility promotes protein aggregation and precipitation, which poses significant challenges for antibody functionality, stability, pharmacokinetics, bioavailability, and pharmaceutical formulation. Poor solubility and high precipitation propensity complicate large-scale manufacturing, purification, and storage processes, ultimately increasing production costs and the risk of immunogenicity (164, 165). A major determinant of protein solubility is its amino acid sequence, which defines the molecule’s three-dimensional structure, charge distribution, and potential sites for post-translational modifications. For example, hydrophobic residues tend to promote protein aggregation, whereas glycosylation can stabilize protein folding and enhance structural stability (166), while charged residues generally contribute to improved solubility. In addition, formulation parameters such as pH, ionic strength, and the presence of excipients, can further influence protein solubility and stability. To address solubility-related challenges, high-throughput screening techniques are increasingly used to identify antibody candidates with favorable solubility profiles. These strategies include sequence optimization, glycoengineering, formulation refinement, and targeted structural modifications, all of which contribute to improved biophysical properties and overall developability of antibody therapeutics (126).

In recent years, AI-driven predictive models have been developed to assess and improve antibody solubility and reduce precipitation tendencies at early stages of antibody design. In addition, several computational tools can estimate precipitation risk by predicting related properties such as aggregation propensity, intermolecular charge distribution, molecular interactions, and intrinsic solubility. For example, SOLpro is a computational tool designed to predict protein solubility based on amino acid sequence features. For example, SOLpro accurately predicts protein solubility during overexpression using amino acid sequences as input data. In addition, it provides mutation suggestions to improve the solubility of antibody candidates, thereby influencing decision-making in therapeutic antibody development (167). SOLpro is structured as a two-stage Support Vector Machine (SVM) model. In the first stage, a trained classifier analyzes sequence features, while the second stage produces the final solubility prediction. The model’s performance was validated through repeated 10-fold cross-validation, demonstrating robust predictive accuracy (167). Another widely used protein engineering tool is FoldX, which suggests specific amino acid mutations to enhance antibody solubility while preserving structural stability and functional efficacy. FoldX enables the rational optimization of antibody candidates, supporting both developability and biotherapeutic performance (168).

CamSol method predicts protein solubility under varying pH conditions with a level of accuracy comparable to experimental methods (169). CamSol detects the hydrophobic patches and analyzes the physicochemical properties of the candidate antibody and suggests specific mutations to enhance solubility while maintaining biological activity. This approach was successfully applied to a single-domain antibody targeting Alzheimer’s disease, accurately predicting solubility changes resulting from specific mutations (170).

Furthermore, the Protein Solubility Predictor (PaRSnIP) is a sequence-based method that employs a gradient boosting machine algorithm, integrating both sequential and structural features of the antibody candidate to predict its solubility with high accuracy, particularly for high-concentration formulations (171). Another machine learning model, PROSO II, has also been developed for protein solubility prediction. It was trained on a continually expanding dataset incorporating experimental solubility data, improving its predictive reliability and applicability in antibody design and development (172). Additionally, solPredict utilizes information derived from amino acid sequences in combination with ESM1b protein language modeling to predict the solubility of antibody candidates, particularly for high-concentration biotherapeutic formulations (173).

The ESM1b model was developed to predict the functional impact of amino acid substitutions on proteins. Using a protein language model trained on a vast dataset of natural protein sequences, ESM1b can evaluate how individual mutations affect antibody solubility, stability, and overall function. The model assesses the influence of each amino acid by comparing the probability distributions of wild-type and mutant residues, providing insights into how disruptive a given mutation may be to structural or functional integrity. Benchmark analyses demonstrated that ESM1b’s predictions correlate strongly with experimental data and clinical classifications of pathogenicity. Furthermore, the model can identify isoform-specific deleterious variants that are difficult to detect using alignment-dependent methods. Although its predictions are statistical and require biological validation, ESM1b offers a scalable, alignment-free framework for variant effect prediction, delivering valuable guidance in therapeutic antibody design and optimization (174).

Another deep learning–based tool, DeepSol, predicts protein solubility directly from amino acid sequences using a convolutional neural network architecture. It extracts structural and physicochemical features from the input sequence to generate accurate predictions (175). DeepSol is available in three versions; DeepSol S1, DeepSol S2, and DeepSol S3; each with progressively enhanced performance and precision. Finally, Quantitative Structure–Activity Relationship (QSAR) models apply machine learning techniques to estimate monoclonal antibody solubility. These models first construct predictive frameworks based on amino acid sequence descriptors and selected physicochemical features, followed by a testing and validation phase to generate the final solubility predictions (176).

Predicting antibody solubility and precipitation remains challenging as these factors are highly controlled by a complex interplay between sequence composition, 3D structures, intermolecular interactions, development and formulation conditions. Future integration of multiple data layers, including structural modeling, molecular dynamics simulations, and large experimental developability datasets may be of great help. Such integrative frameworks could enable more reliable early-stage antibody candidates screening for reducing downstream development risks and improving the efficiency of biotherapeutic discovery.

#### 3.4.2 Aggregation and viscosity

During antibody development, aggregation is a frequent relevant challenge that can lead to loss of stability and functionality of the therapeutic protein, as well as undesirable immune responses (177, 178). In addition, aggregation propensity and poor solubility are among the main causes of developmental and manufacturing difficulties encountered in therapeutic antibody production. The tendency of an antibody to aggregate is primarily influenced by its amino acid sequence, hydrophobicity, hydrophilicity, and surface charge. These molecular features determine the nature of intermolecular interactions, which may promote aggregation, particularly under high-concentration formulation conditions (179, 180).

Both aggregation and viscosity are key developability-related biophysical parameters during antibody design and manufacturing. Aggregation often arises from self-association driven by hydrophobic or electrostatic interactions, environmental stressors, or post-translational modifications, ultimately leading to reduced efficacy, increased immunogenicity, and manufacturing complications. Furthermore, protein aggregation directly contributes to increased solution viscosity, which is a critical factor affecting high-concentration formulations and administration feasibility.

Aggrescan3D (A3D) predicts the aggregation propensity of a protein based on analysis of its three-dimensional structure. It enhances protein solubility by identifying aggregation-prone regions and suggesting point mutations in these areas, followed by in silico screening of variants to select those that most effectively improve solubility without compromising antibody stability. The updated version, A3D 2.0, offers improved usability, visualization, and the ability to process large multimeric proteins. It integrates several complementary tools: CABS-flex for flexibility simulations, FoldX for stability and structural integrity assessments, and an automated mutation module for minimizing aggregation propensity. This integrated approach improves solubility while preserving functionality and has been successfully validated across various proteins (181).

The High Viscosity Index (HVI) is a machine learning model designed for rapid screening and identification of high-viscosity antibody candidates, particularly at high formulation concentrations. It analyzes the distribution of hydrophobic and hydrophilic regions within the variable domain and employs logistic regression and decision tree algorithms to generate predictive outputs. The HVI model was validated using 27 FDA-approved monoclonal antibodies and demonstrated high accuracy in viscosity classification (182).

These computational tools enable efficient, high-throughput screening and support the design and optimization of stable, high-concentration antibody formulations for clinical applications. Future developments will likely benefit from integrating structural modeling, large experimental developability datasets, molecular simulation techniques and databases of already approved antibodies to better represent the physicochemical environment of antibody formulations.

### 3.5 Antibody structure predictions and design

Prediction of protein structure including antibodies is essential for understanding their cellular function and potential interactions with other biomolecules. Significant progress has been achieved in computational protein structure prediction, enabling the optimization of binding affinity, specificity, stability, and other developability-related attributes, **Table 6** (183, 184). For instance, DeepAb is a deep learning tool that accurately predicts the three-dimensional (3D) structure of an antibody’s variable region. It estimates inter-residue geometric distances and orientations, which are then used to construct a precise 3D model of the candidate antibody. In addition to structure prediction, DeepAb can suggest stabilizing mutations, thereby facilitating protein optimization and supporting therapeutic antibody design and development (119).

**Table 6:** AI-based tools and computational approaches for antibody structure prediction and modeling.

AI tool	Principle	Site	Advantages	Limitations
Rosetta Antibody Design (RAbD)	heuristic optimization with Monte Carlo sampling model	–	CDR template sampling; docking + affinity optimization	Computationally intensive; template dependence
AlphaFold2	sequence-based DL	https://alphafold.ebi.ac.uk/	Near-atomic accuracy; deep biological representations, rained on PDB-scale datasets	Limited antibody-specific refinement
AlphaFold3	multimolecular joint-structure modeling DL	https://alphafold.ebi.ac.uk/	Extends structure prediction to complexes	Computationally demanding
IgFold	sequence-based protein language model DL	https://cosmic-cryoem.org/tools/igfold/	Fast; confidence estimates; nanobody compatible	Approximate side-chain modeling
ImmuneBuilder	sequence-based DL	https://neurosnap.ai/service/Immune%20Builder	Fast; includes error estimation	Limited antigen-context modeling
AbDiffuser	sequence and structure-based DL	https://arxiv.org/abs/2308.05027	Uses physics-informed diffusion model and equivariant neural network, sequence–structure generation	model complexity
DeepSCAb	sequence-based DL	https://github.com/Graylab/DeepSCAb	dual-module geometry for backbone and side-chain prediction	model complexity

Additionally, Rosetta Antibody Design (RAbD) is a structure-based, modular tool for in silico antibody modeling and engineering. RAbD predicts antibody structures and enhances antibody–antigen binding by sampling combinations of CDR templates from a canonical CDR cluster database, followed by refinement through sequence, length, and conformational optimization. The method employs Monte Carlo algorithms for CDR loop sampling and optimization. Moreover, RAbD performs antibody–antigen docking, allowing the prediction and improvement of binding interactions and complex stability, thereby supporting rational antibody engineering for enhanced therapeutic performance (149, 185, 186).

AlphaFold is a highly accurate sequence-based protein structure predictor that leverages the vast datasets of known protein sequences and structures. Its core consists of a neural network–based machine learning architecture trained on the Protein Data Bank. The neural network design of AlphaFold was developed to align with the modern understanding of complex protein biology, incorporating well-defined interlayer communication mechanisms that became the key to its exceptional accuracy and predictive performance (187). AlphaFold2 can predict protein structures with near-atomic accuracy, even for targets without any known homologous structures (187). A more advanced version, AlphaFold3, further advances the model’s capabilities, providing improved accuracy for predicting protein structures and protein–ligand interactions. Moreover, AlphaFold3 can model joint structures of complex biological assemblies, including nucleic acids, small molecules, ions, post-translationally modified residues, and antibodies, enabling a more comprehensive understanding of molecular interactions (188).

AbPredict is a computational tool that models the antibody variable domain structure without relying exclusively on homologous structural templates. Instead, it utilizes Monte Carlo sampling to explore a wide range of backbone conformations and side-chain orientations, generating diverse candidate structures. While the method may encounter challenges with rare loop lengths, it offers a fully automated antibody modeling workflow that operates approximately 20 times faster than traditional template-based approaches while maintaining robust accuracy (189).

Additionally, IgFold is a rapid deep learning–based model for antibody structure prediction that relies solely on the amino acid sequence as input. It integrates a pre-trained language model trained on more than 558 million antibody sequences, which provides contextual embeddings that are subsequently processed through graph neural networks to generate backbone atomic coordinates. IgFold also delivers per-residue error estimations, offering confidence metrics for its predictions. The method is compatible with both conventional antibodies and nanobodies, demonstrating good accuracy and computational efficiency in large-scale antibody repertoire modeling (184).

ImmuneBuilder represents a suite of deep neural network tools for modeling antibody and T-cell receptor structures. It includes ABodyBuilder2 for traditional antibodies, NanoBodyBuilder2 for nanobodies, and TCRBuilder2 for T-cell receptor prediction. ImmuneBuilder provides fast and reliable performance, accompanied by error estimation metrics to assess prediction confidence (190). AbDiffuser is an innovative generative framework capable of simultaneously predicting both the amino acid sequence and 3D structure of antibody candidates for specific antigens. It employs a physics-informed diffusion-based approach combined with an equivariant neural architecture (APMixer) to accurately model both backbone and side-chain conformations. Notably, AbDiffuser has been successfully applied to design antibody candidates against HER2, several of which demonstrated high binding affinity during experimental validation (191). DeepSCAb is another deep learning tool that predicts both backbone geometry and side-chain conformations of antibody candidates through a dual-module architecture. The first module infers inter-residue distances and orientations, while the second estimates side-chain dihedral angles, providing a more complete structural prediction pipeline (192).

Protein structure databases are essential for training predictive models that estimate antibody structure, as well as other attributes related to development and manufacturing. The application of AI-based generative models can help address crystallographic challenges, particularly when structural limitations arise during validation and discovery processes (193). Improving both the quality and quantity of such datasets can significantly enhance the performance and predictive accuracy of AI models. In this context, some generative AI tools are capable of proposing candidate crystal structures and predicting their physicochemical properties (194). These approaches aim to identify low-energy configurations using algorithms such as USPEX (195), CALYPSO (196), and the minima hopping method (197), among others. Moreover, modern generative AI models trained on large structural databases can suggest novel and plausible crystal structures in a more time- and cost-efficient manner while maintaining good predictive accuracy (198, 199). Such algorithms are particularly valuable in antibody research, where they can support the design of new antibodies or the optimization of existing structures to improve antigen binding and interactions with target molecules (200). As mentioned in our article AI-based tools and methods, along with many others currently under development, hold great promise for the future of antibody design and development processes, enabling a more time- and cost-efficient approach for diverse clinical applications.

## 4 Discussion and conclusion

Monoclonal antibodies have become indispensable in clinical and scientific applications due to their efficacy and high target-specificity (201). Recognizing their growing relevance, we aimed in this review to provide a structured comprehensive overview of technologies used for designing, developing, and manufacturing biotherapeutics.

The earliest widely adopted technology for antibody generation was the hybridoma technique. Although highly foundational, hybridoma still has limitations, including relatively low efficiency and frequent loss of natural heavy- and light-chain pairing. Alongside hybridoma technology, several other traditional methods have also been applied, such as B-cell immortalization, display systems, gene editing approaches, and transgenic animals (202–204). Despite their usefulness, these techniques share significant constraints. Most notably, they are time-consuming; antibody identification, selection, and optimization may require years of intensive laboratory work. Furthermore, the overall quality of the output heavily depends on the individual expertise of the operator, making the process highly subjective and vulnerable to human error. Another major challenge is the difficulty of isolating antibodies that bind precisely to a defined epitope on the target antigen. Screening campaigns may yield antibodies that recognize unintended epitopes, resulting in diminished or entirely absent therapeutic effect. Additionally, traditional methods do not ensure that selected antibodies possess favorable developability properties. There is no guarantee that a candidate antibody can be efficiently manufactured, formulated at clinically relevant concentrations, or delivered to patients in a stable and effective form. Downstream optimization steps, including affinity maturation and refinement of antigen–antibody interactions, typically rely on methods such as X-ray crystallography or NMR spectroscopy, both of which are highly resource-intensive and need complex preparation steps. In summary, traditional monoclonal antibody development methods face persistent challenges related to time, cost, labor, and output quality. They often yield limited quantities of product and offer no assurance that the resulting antibodies meet the required therapeutic or developability criteria. These constraints have driven the field toward more advanced, efficient, and objective technological solutions, as discussed throughout the review. Additionally, with the increasing problem of treatment resistance and inefficacy, the demand for new monoclonal antibodies has grown substantially. This growth created an urgent need for faster, easier, and more effective developmental workflows. In response, artificial intelligence has emerged as a powerful enabler. Since its introduction into the field, dependence on AI-based tools and computational instruments has steadily increased especially with the progress of science and technology particularly in genome sequencing, structural analysis, and the establishment of large biological databases.

The revolution of AI-based tools is now being applied across all areas of scientific and industrial practice. Immunoinformatics, in particular, has transformed the process of antibody design and development. Modern computational tools address every aspect of these workflows. The core principle underlying AI methods is the use of trained machine learning, deep learning, and artificial neural network algorithms on databases containing sequential or structural information. Earlier tools tended to focus on optimizing only one attribute at a time, such as epitope structure or CDR structure. More recent tools are often hybrid in nature, integrating data from various databases. Importantly, they are now capable of optimizing multiple attributes simultaneously. For example, a single tool can identify an epitope or CDRs while also suggesting affinity-enhancing mutations, maintaining developability profiles, and reducing immunogenicity. Integration of artificial intelligence into immunopharmaceutical research represents a transformative step in biotechnology. Over the past decade, the application of computational modeling algorithms has reshaped traditional workflows for monoclonal antibody design, engineering, and optimization. AI-driven methodologies now offer accelerated discovery pipelines, refined molecular design, CDR sequence optimization, more accurate prediction of antibody–antigen interactions as well as therapeutic efficacy, and safety (7, 90, 205).

**Figure 3:**
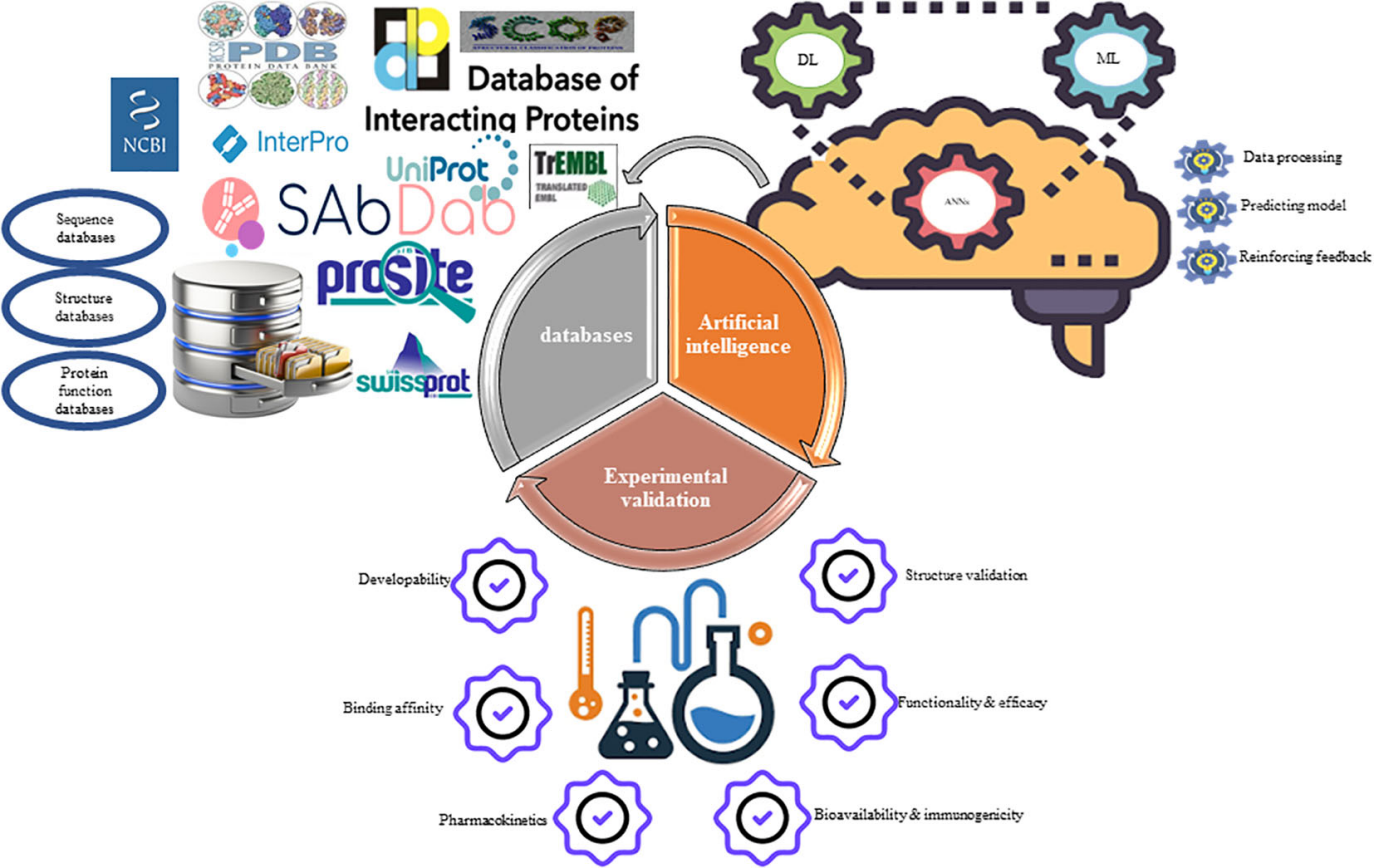
The interplay between databases, AI tools, and experimental validation.

The relationship between in silico methods and experimental data is bidirectional and mutually reinforcing, **Figure 3**. On one hand, computational tools rely on large volumes of information to train their algorithms and models, the more experimental data accumulated, the larger and more diverse the available datasets become. These larger datasets, in turn, improve the training of language models and computational algorithms. On the other hand, as the performance of these tools improves, they become increasingly capable of processing and interpreting complex data, which helps refine and expand existing datasets. This is particularly valuable for structural methods, where enhanced data analysis can directly contribute to solving current challenges in the field. Beyond design and development, AI applications in bioprocessing and production optimization are also beginning to be widely applied. Machine learning models are being developed to automate post-design stages of antibody manufacturing, including real-time monitoring and optimization of cell-culture conditions. These models can analyze signals from sensors and process-control systems, enabling continuous assessment of culture performance and prediction of productivity (206, 207).

Despite their advanced capabilities, speed, and efficiency, AI tools still face limitations that require further refinement. Current challenges include predictive inaccuracies, high computational demands, and the scarcity of comprehensive, high-quality datasets. AI models also struggle with generalization and transferability across diverse biological systems (205, 208, 209). For example, AI-generated *de novo* antibody sequences may reduce conformational flexibility, potentially compromising affinity and functionality. In some cases, heavy dependence on predefined templates and biased training data may limit manufacturability or overlook essential developability attributes. Additionally, computational frameworks that optimize a single molecular property risk inadvertently impair other attributes such as stability, immunogenicity or expression yield. However, many modern antibody-design models have begun to overcome these problems by explicitly integrating manufacturability constraints alongside efficacy and specificity targets (205). Another persistent challenge is the need for large, diverse, and unbiased training datasets. Databases for rare diseases or newly emerging pathogens remain limited, which significantly impacts model accuracy, predictive power, and generalizability (210–212). Overall, the major limitations of existing AI systems stem from inefficiency of prediction, accuracy gaps, and the scarcity of comprehensive data resources. Integrating multi-omics datasets, including genomic, proteomic, and transcriptomic information as well as data from empirical structural biology has recently helped enhance predictive robustness by providing richer, more biologically representative inputs for model training.

In conclusion, traditional laboratory methods remain essential for validating AI-generated predictions, **Figure 3**. A combined approach; integrating computational modeling with experimental testing; represents the optimal strategy for designing and developing new monoclonal antibodies. Laboratory assays are particularly necessary for evaluating critical properties such as stability, solubility, immunogenicity, and binding affinity. When effectively integrated, AI tools and experimental workflows complement one another, reducing time and labor requirements while enhancing accuracy and precision (138, 213, 214).

The continued evolution of artificial intelligence in antibody engineering is expected to drive the development of more integrated, autonomous, and efficient workflow systems. As high-throughput experimental data accumulate, AI models will gain access to increasingly diverse and informative training datasets, supporting the generation of novel functional antibodies (215). Deep generative diffusion models are beginning to bridge the gap between sequence-level data and atomic-resolution structural predictions, showing strong potential for optimizing CDR structures, generating novel antibody sequences, and modeling three-dimensional molecular conformations (216–219). Further improvements in AI platforms will depend on deeper integration of interdisciplinary scientific capabilities and the development of multi-objective optimization frameworks capable of balancing efficacy, stability, affinity, and manufacturability in designed antibody candidates. Techniques such as self-supervised learning and generative modeling offer promising solutions for overcoming data scarcity, thereby enhancing AI’s capacity to generate high-quality predictions. Additionally, advancements in explainable AI can improve interpretability, strengthen model reliability, and increase confidence in computational outputs. Ultimately, the integration of advanced computational design approaches with rigorous experimental validation alongside AI-driven bioprocess control will be essential for addressing current limitations in both fields and accelerating the development of next-generation antibody therapeutics.
